# Induction of *MDR1* gene expression by anthracycline analogues in a human drug resistant leukaemia cell line

**DOI:** 10.1038/sj.bjc.6990133

**Published:** 1999-02

**Authors:** X F Hu, A Slater, D Rischin, P Kantharidis, J D Parkin, J Zalcberg

**Affiliations:** Trescowthick Laboratory, Peter MacCallum Cancer Institute, Melbourne, Australia; Division of Pathology, Austin and Repatriation Medical Centre, Melbourne, Australia

**Keywords:** drug resistance, induction of *MDR1* expression, anthracycline analogues, Pgp expression, drug accumulation

## Abstract

The effects of 4-demethoxydaunorubicin (idarubicin, IDA) and MX2, a new morpholino-anthracycline, on up-regulation of the *MDR1* gene in the low-level multidrug resistant (MDR) cell line CEM/A7R were compared at similar concentrations (IC_10_, IC_50_and IC_90_) over a short time exposure (4 and 24 h). The chemosensitivity of each drug was determined by a 3-day cell growth inhibition assay. Compared with epirubicin (EPI), IDA and MX2 were 17- and eightfold more effective in the CEM/A7R line respectively. No cross-resistance to 5-FU was seen in the CEM/A7R line. Verapamil (5 μM) and PSC 833 (1 μM), which dramatically reversed resistance to EPI in the CEM/A7R line, had no sensitizing effect on the resistance of this line to MX2, but slightly decreased resistance to IDA. The sensitivity to 5-FU was unchanged by these modulators. The induction of *MDR1* mRNA expression by IDA, MX2 and 5-FU was analysed by Northern blotting and semiquantitatively assessed by scanning Northern blots on a phosphorimager. The relative level of *MDR1* expression was expressed as a ratio of *MDR1* mRNA to the internal RNA control glyceraldehyde-3-phosphate dehydrogenase (GAPDH). IDA, MX2 and 5-FU differentially up-regulated *MDR1* mRNA in the CEM/A7R line in a dose-dependent manner. Both IDA and MX2 induced *MDR1* expression within 4 h. 5-FU up-regulated *MDR1* expression only when drug exposure was prolonged to 24 h. Based on MRK 16 binding, flow cytometric analysis of P-glycoprotein (Pgp) expression paralleled the increase in *MDR1* mRNA levels. For the three anthracyclines, the increase in *MDR1* expression was stable in cells grown in the absence of drug for more than 3 weeks after drug treatment. The induction of *MDR1* expression by 5-FU was transient, associated with a rapid decrease in the increased Pgp levels which returned to baseline 72 h after the removal of 5-FU. This study demonstrates that *MDR1* expression can be induced by analogues of anthracyclies not pumped by Pgp, and that this induction appears to be stable despite a 3-week drug-free period. © 1999 Cancer Research Campaign


*British Journal of Cancer* (1999) **79**(5/6), 831–837
© 1999 Cancer Research Campaign
Article no. bjoc.1998.0133

### Induction of *MDR1* gene expression by anthracycline analogues in a human drug resistant leukaemia cell line

**XF Hu[1], A Slater[1], D Rischin[1], P Kantharidis[1], JD Parkin[2] and J Zalcberg[1]**

[1]Trescowthick Laboratory, Peter MacCallum Cancer Institute, Melbourne, Australia; [2]Division of Pathology, Austin and Repatriation Medical Centre, Melbourne, Australia

**Summary** The effects of 4-demethoxydaunorubicin (idarubicin, IDA) and MX2, a new morpholino-anthracycline, on up-regulation of the *MDR1* gene in the low-level multidrug resistant (MDR) cell line CEM/A7R were compared at similar concentrations ($IC_{10}$, $IC_{50}$ and $IC_{90}$) over a short time exposure (4 and 24 h). The chemosensitivity of each drug was determined by a 3-day cell growth inhibition assay. Compared with epirubicin (EPI), IDA and MX2 were 17- and eightfold more effective in the CEM/A7R line respectively. No cross-resistance to 5-FU was seen in the CEM/A7R line. Verapamil (5 μM) and PSC 833 (1 μM), which dramatically reversed resistance to EPI in the CEM/A7R line, had no sensitizing effect on the resistance of this line to MX2, but slightly decreased resistance to IDA. The sensitivity to 5-FU was unchanged by these modulators. The induction of *MDR1* mRNA expression by IDA, MX2 and 5-FU was analysed by Northern blotting and semiquantitatively assessed by scanning Northern blots on a phosphorimager. The relative level of *MDR1* expression was expressed as a ratio of *MDR1* mRNA to the internal RNA control glyceraldehyde-3-phosphate dehydrogenase (GAPDH). IDA, MX2 and 5-FU differentially up-regulated *MDR1* mRNA in the CEM/A7R line in a dose-dependent manner. Both IDA and MX2 induced *MDR1* expression within 4 h. 5-FU up-regulated *MDR1* expression only when drug exposure was prolonged to 24 h. Based on MRK 16 binding, flow cytometric analysis of P-glycoprotein (Pgp) expression paralleled the increase in *MDR1* mRNA levels. For the three anthracyclines, the increase in *MDR1* expression was stable in cells grown in the absence of drug for more than 3 weeks after drug treatment. The induction of *MDR1* expression by 5-FU was transient, associated with a rapid decrease in the increased Pgp levels which returned to baseline 72 h after the removal of 5-FU. This study demonstrates that *MDR1* expression can be induced by analogues of anthracyclies not pumped by Pgp, and that this induction appears to be stable despite a 3-week drug-free period.

**Keywords:** drug resistance; induction of *MDR1* expression; anthracycline analogues; Pgp expression; drug accumulation

Drug resistance is a common problem in acute leukaemia (Mickenna et al, 1997). Patients often relapse with unresponsive disease after an initial response to treatment with cytotoxic drugs (Rothenberg et al, 1989). The expression of P-glycoprotein (Pgp) encoded by the *MDR1* gene (Deuchars and Ling, 1989), is a well-known mechanism of multidrug resistance in relapsed acute leukaemia (Shustik et al, 1995; Bosch and Croop, 1996). The level of *MDR1* gene expression in acute leukaemia is commonly increased after chemotherapy (Grogan et al, 1993; Nooter and Sonneveld, 1994). In addition, there appears to be a direct correlation between expression of the *MDR1* gene de novo and outcome (survival) in this disease (Poeta et al, 1996).

Although the mechanisms underlying the effect of cytotoxic drugs on *MDR1* gene expression and in turn the multidrug resistant (MDR) phenotype remain poorly understood, the acquisition of Pgp-mediated drug resistance during chemotherapy is usually thought to be due to the selection of drug-resistant cells (Chaudhary and Roninson, 1993; Gekeler et al, 1994; Manzano et al, 1996). Sikic and colleagues have clearly demonstrated this in a cell line model (Beketic-Oreskovic et al, 1994; Chen et al, 1994; Dumontet et al, 1996), under conditions in which up-regulation of Pgp was prevented. Although several attempts have been made to demonstrate that Pgp expression can be up-regulated, these studies have generally required long exposure times (72 h or more). Consequently, the selection of Pgp-expressing cells could not be ruled out from these studies (Manzano et al, 1996).

However, we have demonstrated that a rapid induction of the *MDR1* gene can occur after the exposure of cells to anthracyclines in a human MDR cell line (CEM/A7R) known to express low-level *MDR1* mRNA and P-glycoprotein (Hu et al, 1995). The induction of *MDR1* was dose and time related and correlated with an increase in Pgp expression and drug resistance. The rapid increase in *MDR1* gene expression after exposure to daunorubicin or epirubicin strongly supported the previous findings that the *MDR1* gene promoter was activated by anti-cancer agents (Kohno et al, 1989). Similar effects have been reported in rodent cell lines (Chin et al, 1990; Fardel et al, 1997). This induction of *MDR1* gene expression may have an important role in our understanding and treatment of drug-resistant tumours, as it can be prevented by cyclosporin A (CyA) and its analogue PSC 833 (Hu et al, 1996).

Alternative approaches designed to overcome the MDR phenotype include the use of new lipid-soluble compounds that are not substrates for Pgp (De Vries et al, 1990; Michieli et al, 1993). However, the effect of these agents on *MDR1* gene expression is unknown. The present study was designed to investigate whether two new lipid-soluble anthracyclines, 4-demethoxydaunorubicin (idarubicin, IDA) and MX2, a new morpholino-anthracycline,

*Received 22 April 1998*
*Revised 6 July 1998*
*Accepted 7 July 1998*

*Correspondence to:* JR Zalcberg, Division of Haematology and Medical Oncology, Peter MacCallum Cancer Institute, Locked Bag 1, A'Beckett Street, Melbourne, Vic. 3000, Australia

**Table 1** Chemosensitivity of the CEM/A7R and parental CCRF-CEM cell lines to EPI, MX2, IDA and 5-FU

| | CEM/A7R | | | CCRF-CEM | | |
|---|---|---|---|---|---|---|
| Drug | $IC_{10}$ (μg ml$^{-1}$) | $IC_{50}$ (μg ml$^{-1}$) | $IC_{90}$ (μg ml$^{-1}$) | $IC_{10}$ (μg ml$^{-1}$) | $IC_{50}$ (μg ml$^{-1}$) | $IC_{90}$ (μg ml$^{-1}$) |
| EPI | 0.103 ± 0.002 | 0.373 ± 0.041 (5.4) | 0.965 ± 0.035 | 0.008 ± 0.001 | 0.075 ± 0.003 | 0.128 ± 0.02 |
| MX2 | 0.020 ± 0.002 | 0.045 ± 0.011 (3.2) | 0.107 ± 0.009 | 0.004 ± 0.001 | 0.014 ± 0.003 | 0.043 ± 0.003 |
| IDA | 0.003 ± 0.001 | 0.022 ± 0.008 (3.1) | 0.080 ± 0.025 | 0.002 ± 0.001 | 0.007 ± 0.001 | 0.016 ± 0.002 |
| 5FU | 0.180 ± 0.04 | 0.51 ± 0.22 (1.0) | 2.60 ± 0.230 | 0.20 ± 0.008 | 0.454 ± 0.122 | 2.615 ± 0.235 |

$IC_{10}$, $IC_{50}$ and $IC_{90}$ were determined as described in the Materials and methods section. Results are expressed as the average concentration (μg ml$^{-1}$) ± standard deviation (calculated from three experiments). Each experiment was performed in triplicate. Figures in parentheses represent relative resistance (see 'Materials and methods').

were able to up-regulate *MDR1* gene expression compared with the classic anthracycline epirubicin (EPI).

### MATERIALS AND METHODS

### Materials

EPI and IDA and verapamil were obtained commercially from Farmitalia (Melbourne, Australia). MX2 was a gift from Kirin Brewery Company (Japan). Verapamil was dissolved in 0.9% saline solution. PSC 833 was obtained from Sandoz Pharma (Basel, Swizerland) and initially dissolved in absolute alcohol before being diluted in RPMI-1640 to give a stock solution of 0.5 mg ml$^{-1}$ (the final ethanol concentration was 35%). RPMI-1640 was purchased as a powder (Gibco) and supplemented with 10% fetal calf serum (FCS) (Trace Biosciences, Melbourne, Australia), gentamicin (80 μg ml$^{-1}$), minocycline (1 μg ml$^{-1}$), Hepes (20 mM), sodium bicarbonate (0.21%) and glutamine (0.8 mM). Monoclonal antibody (mAb) MRK 16 to Pgp was generously provided by Dr Takashi Tsuruo (Division of Experimental Chemotherapy, Japanese Foundation for Cancer Research). A fluorescein-labelled $F(ab)_2$ fragment of sheep anti-mouse IgG was purchased from Silenus Laboratories (Melbourne, Australia). The cDNA probe pHDR5A was a gift from Dr M Gottesman and Dr Ira Pastan (Laboratory of Molecular Biology, National Institutes of Health, Bethesda, MD, USA). The cDNA glyceraldehyde-3-phosphate dehydrogenase (GAPDH) was a gift from Dr Mark Ross (Ludwig Institute for Cancer Research, Melbourne, Australia). Propidium iodide was purchased from Sigma Chemical (St Louis, MO, USA).

### Cell lines and drug treatment

The studies were carried out in a variant human T-cell leukaemia MDR cell line, CEM/A7R. This line was derived from a classic MDR cell line CEM/A7, selected for low-level doxorubicin (DOX) resistance by stepwise selection of the parental line, CCRF-CEM, cultured in increasing concentrations of DOX (Zalcberg et al, 1994). The resistant line CEM/A7 was maintained in conditioned medium containing 0.07 μg ml$^{-1}$ of DOX. The variant line (now stable for over 3 years) was established by culturing the CEM/A7 cells in the absence of DOX before being subcloned and designated as the CEM/A7R line. This line was not exposed to DOX or other Pgp substrates except in the specific experiments detailed below. At the time of these experiments, all lines were mycoplasma-free based on the mycoplasma TC Rapid kit (Gen-Probe, San Diego, CA, USA).

The CEM/A7R cells in exponential growth phase were collected 2 days after subculture. The cells were washed, counted and resuspended in 20 ml of fresh medium to a total cell number of $5 \times 10^6$–$1 \times 10^7$. The cells were treated with various concentrations of EPI, IDA and MX2 for 4–24 h, then harvested and washed three times with cold phosphate-buffered saline (PBS) for the analysis of *MDR1* gene expression. Cell viability was determined (after staining by trypan blue) using phase-contrast microscopy to detect cells of abnormal size or granularity. Non-viable cells were excluded from flow cytometric analysis by propidium iodide staining.

### Growth assays

The sensitivity of each of the cell lines to a variety of chemotherapeutic drugs was determined by a standard growth inhibition assay (Tsuruo et al, 1981). Briefly, after determining cell viability, $2 \times 10^5$ of the tested cells were exposed to varying concentrations of each tested drug in the presence or absence of verapamil (Vp) or PSC 833 in 12-well plates. The cells were incubated at 37°C in a humidified chamber containing 5% carbon dioxide in air for 3 days and counted using an automated coulter counter (Hu et al, 1990*a*). Results are expressed as the increase in cell number of drug-exposed cells as a percentage of the increase in untreated control cells. The $IC_{10}$, $IC_{50}$ and $IC_{90}$ for each drug were determined by calculating the drug concentration required to inhibit cell growth by 10%, 50% and 90%. Relative resistance represents the ratio of the $IC_{50}$ of the resistant CEM/A7R cell line compared with the parental CCRF-CEM cell line.

### RNA extraction and Northern blot analysis

RNA was isolated by the guanidinium thiocyanate method described by Chomczynski and Sacchi (1987). Twenty micrograms of total cellular RNA was size fractionated on a 1.5% agarose gel containing 2.2 M formaldehyde and transferred onto nylon filters (Hybond-N, Amersham, UK) for *MDR1* hybridization. The filters were probed with the plasmid pHDR5A containing a 1.4-kb *MDR1* cDNA (Ueda et al, 1987) and then reprobed with a $^{32}P$-labelled GAPDH cDNA for normalization. The pHDR5A probe predominantly recognizes the *MDR1* gene under the high stringency conditions used in this study. The filters were prehybridized overnight at 42°C in hybridization buffer containing 50% formamide, 5 × SSPE (1 × SSPE containing 0.15 M sodium chloride, 0.001 M sodium dihydrogen phosphate and 0.001 M EDTA), 5 × Denhardt's solution,

 © Cancer Research Campaign 1999

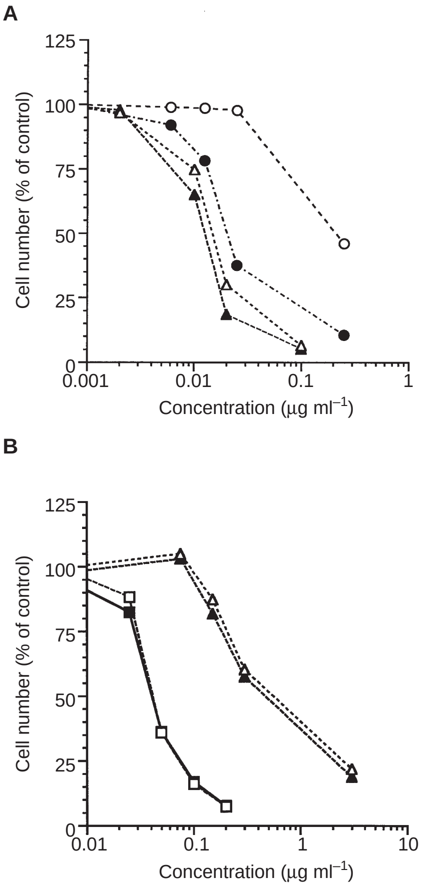

**Figure 1** (**A**) The effect of PSC 833 on chemosensitivity of CEM/A7R cells as a function of increasing concentrations of EPI or IDA. Growth curves in the absence (○–○) or presence of 0.1 μM PSC 833 (●–●) as a function of EPI concentrations and in the absence (△–△) or presence (▲–▲) of 1 μM PSC 833 as a function of IDA concentrations. (**B**) The effect of PSC 833 on the chemosensitivity of CEM/A7R cells as a function of increasing concentrations of 5-FU or MX2. Growth curves in the absence (△–△), (□–□) or presence (▲–▲), (■–■) of 1 μM PSC 833 as a function of 5-FU and MX2 concentrations respectively

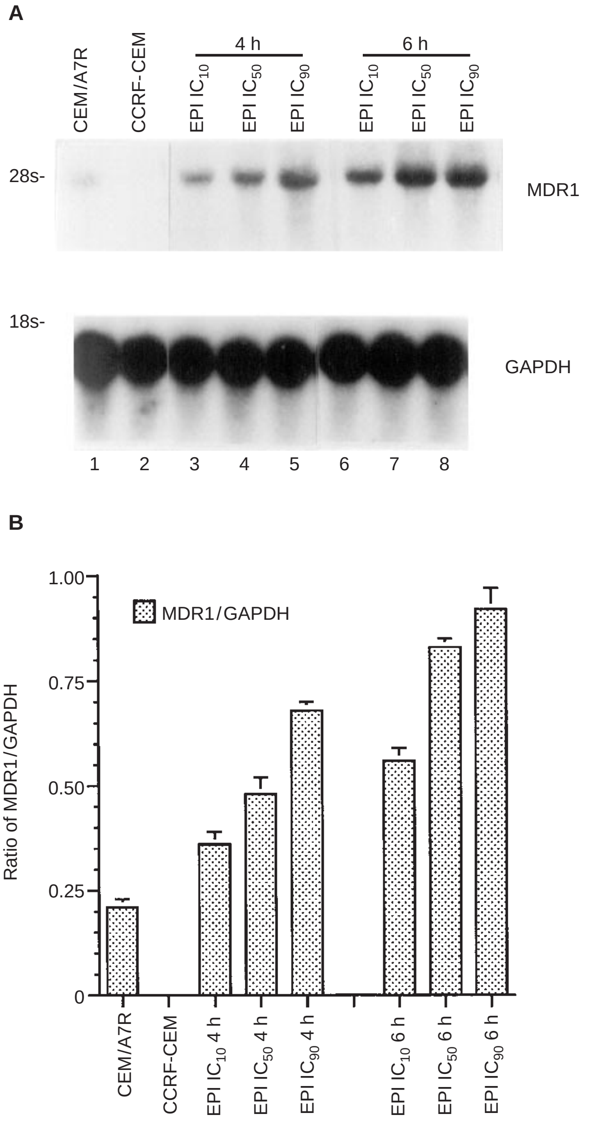

**Figure 2** (**A**) Effect of increasing doses of EPI over a short time on up-regulation of *MDR1* in the CEM/A7R line. Cells in exponential growth phase were treated with (or without) 0.1 ($IC_{10}$), 0.5 ($IC_{50}$), 1.0 ($IC_{90}$) μg ml$^{-1}$ of EPI for 4–6 h. RNA was extracted and analysed by Northern blotting as described in the Materials and methods section. The migration of 28s and 18s ribosomal RNA are indicated. The *MDR1* mRNA level of the parental, sensitive CCRF-CEM line represented a negative Pgp control. (**B**) The Northern analysis shown in **A** was scanned on a phosphorimager using Image Quant Software (Molecular Dynamics). The results were expressed as the ratio of *MDR1* to GAPDH after signals were normalized with respect to the CCRF-CEM signal. Columns, means of triplicate slot blot analysis; bars give the standard deviation. Similar dose– and time–response patterns were seen in a repeat experiment

0.5% sodium dodecyl sulphate (SDS) and 1% skimmed milk powder. Hybridization was carried out in the same buffer. The pHDR5A and GAPDH cDNAs were randomly primed with [α-$^{32}$P]-dCTP. Labelled cDNA ($10^6$ c.p.m.) was added to each millilitre of hybridization buffer. The filters were washed sequentially in 2 × SSPE with 0.1% SDS at 42°C for 15 min, 1 × SSPE with 0.1% SDS at 65°C for 30 min and finally 0.1 × SSPE with 0.1% SDS at room temperature for 15 min. The filters were then exposed to radiographic film at –70°C using intensifying screens, or radioactive signals were quantitated by scanning on a phosphorimager using Image Quant software (Molecular Dynamics, Melbourne, Australia).

### Pgp expression

Flow cytometry was used to measure Pgp expression. Cells were collected and washed three times in medium containing 10% FCS. MRK16, a mAb to an external epitope of Pgp (final concentration 10 μg ml$^{-1}$), was added to cells at room temperature (RT) for 20 min. A non-specific murine mAb ($IgG_{2a}$, Becton Dickinson, Sydney, Australia) was used as the isotype control. After a further three washes, cell pellets were resuspended in the same volume of phosphate-buffered saline (PBS) containing 10 μl of a 1:10 dilution of a fluorescein-conjugated $F(ab')_2$ fragment of sheep anti-mouse IgG antibody (Silenus Laboratories) for 20 min at RT in the dark. Cells were washed once again (×3) and then analysed in a FACScan flow cytometer (Becton Dickinson). Mean fluorescence intensity was recorded for each tested population (after correcting for non-specific binding) to provide an estimate of relative MRK-16 binding.

© Cancer Research Campaign 1999

### [³H]Daunomycin accumulation

Changes in [³H]daunomycin accumulation were used as a functional assay of Pgp (Hu et al, 1990*b*). Cells were washed three times with PBS and resuspended in fresh medium for 1 h at 37°C in a humidifier before performing drug accumulation studies. The cells were adjusted to a concentration of $5 \times 10^6$ ml$^{-1}$ and viability assessed with trypan blue. Cells were added to 96-well plates to give a final number of $5 \times 10^5$ cells per well and incubated at 37°C with tracer amounts of [³H]daunomycin (final concentration $1.85 \times 10^4$ Bq ml$^{-1}$, 0.05 µg ml$^{-1}$). The cells were harvested onto glass-fibre filters at designated times with an automated cell harvester (Cambridge Technology). The filter papers were dried and dissolved in 5 ml of a liquid scintillation cocktail (Ultima Cold, Packard) before radioactivity was measured. All assays were performed in triplicate.

### Statistics

Analysis of variance was used to compare intracellular levels of [³H]daunomycin after exposure of cells to various experimental conditions.

### RESULTS

### Cytotoxicity of IDA, MX2 and 5-FU

The relative cytotoxicity of the two lipid-soluble anthracyclines IDA and MX2 was compared with the classic anthracycline epirubicin (EPI), as well as the unrelated drug 5-FU – a drug not pumped by Pgp. The $IC_{10}$, $IC_{50}$ and $IC_{90}$ for each drug were determined in a 3-day growth inhibition assay (Table 1) in the drug-resistant CEM/A7R and the parental drug-sensitive line CCRF-CEM.

On a weight basis, IDA and MX2 were 17- and eightfold more active than EPI in the drug-resistant line CEM/A7R and 11- and fivefold more active than EPI in the parental line CCRF-CEM, respectively, when comparing doses that inhibited cell growth by 50% ($IC_{50}$). Relative to the drug-sensitive line, there was a fivefold increase in resistance to EPI and a threefold increase in the resistance to IDA and MX2 in the drug-resistant line. As expected, no increase in the resistance to 5-FU was observed in the CEM/A7R line compared with the parental line (Table 1).

PSC 833 (0.1 µM) dramatically reversed the resistance of the CEM/A7R line to EPI (Figure 1A). However, the addition of 5 µM verapamil (data not shown) or 1 µM PSC 833 to the culture medium only slightly increased the sensitivity of this line to IDA (Figure 1A), and had no detectable effect on the sensitivity of the line to MX2 (Figure 1B). Neither modulator had any effect on the sensitivity of the MDR line to 5-FU (Figure 1B).

### Gene expression

The effects of EPI, IDA, MX2 or 5-FU on the induction of the *MDR1* gene in the CEM/A7R line were compared at their respective $IC_{10}$, $IC_{50}$ and $IC_{90}$ concentrations over a 4- or 24-h period of drug exposure. All samples collected for the analysis of *MDR1* gene expression were harvested immediately after drug treatment. The up-regulation of *MDR1* gene expression by each drug was semiquantitatively assessed by scanning Northern blots on a phosphorimager.

We had previously demonstrated that 1.5 µg ml$^{-1}$ of EPI ($IC_{90}$ concentration) induced the expression of *MDR1* mRNA as early as 4 h after such exposure. In the present study, the effect of EPI on the induction of the *MDR1* gene was examined at the $IC_{10}$ and $IC_{90}$ concentrations over a short time period (4–6 h). Up-regulation of *MDR1* mRNA was observed over 4–6 h with a two- to fivefold increase in *MDR1* mRNA noted at 0.1 ($IC_{10}$) and 1.0 ($IC_{90}$) µg ml$^{-1}$ of EPI respectively (Figure 2A and B).

The impact of IDA and MX2 on the induction of *MDR1* gene expression was examined at equieffective concentrations over 4 h. Both drugs induced a similar increase in *MDR1* mRNA levels in a dose-dependent manner (Figure 3). A two- to threefold increase in *MDR1* mRNA was observed at the $IC_{10}$ (0.002, 0.02 µg ml$^{-1}$) and $IC_{50}$ (0.02, 0.05 µg ml$^{-1}$) concentrations of IDA and MX2, and a fivefold increase at the $IC_{90}$ concentrations (0.08, 0.1 µg ml$^{-1}$) of

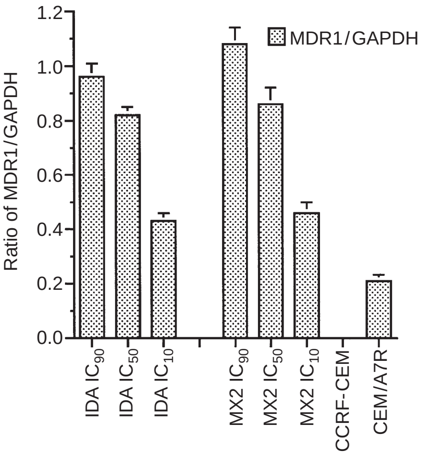

**Figure 3** Effect of IDA or MX2 on induction of *MDR1* gene expression in the CEM/A7R cells after a 4-h exposure. CEM/A7R cells in exponential growth phase were treated with and without IDA at the $IC_{10}$ (0.002 µg ml$^{-1}$), $IC_{50}$ (0.02 µg ml$^{-1}$), and $IC_{90}$ (0.08 µg ml$^{-1}$) levels or MX2 at $IC_{10}$ (0.02 µg ml$^{-1}$) $IC_{50}$ (0.05 µg ml$^{-1}$), $IC_{90}$ (0.1 µg ml$^{-1}$) for 4 h. RNA was then extracted and subjected to Northern blot analysis and scanned on a phosphorimager as described in the Materials and methods section. Results are expressed as the ratio of *MDR1* to GAPDH RNA after signals were normalized with respect to the CCRF-CEM signal. Columns, means of triplicate analyses of Northern blots; bars, s.d. These experiments were repeated on three separate occasions with similar findings. The data presented are representative of one such experiment

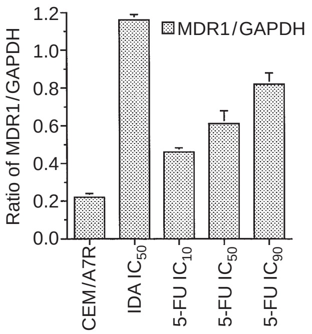

**Figure 4** The effects of 5-FU on the induction of the *MDR1* gene in the CEM/A7R line over a 24-h exposure. Cells in exponential growth phase were treated with (or without) 5-FU at the $IC_{10}$ (0.2 µg ml$^{-1}$), $IC_{50}$ (0.5 µg ml$^{-1}$), $IC_{90}$ (2 µg ml$^{-1}$) or IDA at the $IC_{50}$ level (0.02 µg ml$^{-1}$) as a comparison. The $^{32}$P-labelled *MDR1* and GAPDH bands were scanned on a phosphorimager using Image Quant Software (Molecular Dynamics) and the results are expressed as the ratio of *MDR1* to GAPDH RNA after signals were normalized with respect to the CCRF-CEM signal. Columns, means of triplicate analyses of Northern blots; bars, s.d. Similar results were seen in repeated experiment

© Cancer Research Campaign 1999

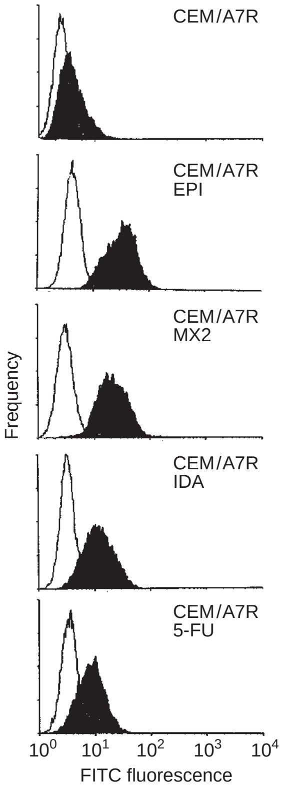

**Figure 5** Flow cytometric analysis of Pgp expression using MRK-16 binding (filled histogram) compared with an $IgG_{2a}$ control (unfilled histogram) in the CEM/A7R line with or without a 24-h exposure to $IC_{90}$ concentrations of EPI (1.0 μg ml$^{-1}$), IDA, MX2 (0.1 μg ml$^{-1}$) or 5-FU (2.0 μg ml$^{-1}$) before measurement of Pgp levels as described in the Materials and methods section

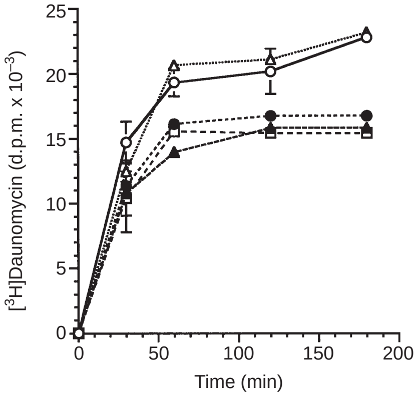

**Figure 6** The intracellular levels of [$^3$H]daunomycin measured in the CEM/A7R cells, 3 weeks after overnight treatment with (or without (○–○), EPI 0.5 μg ml$^{-1}$ (●–●), 0.1 μg ml$^{-1}$ IDA (□–□), 0.1 μg ml$^{-1}$ MX2 (▲–▲) or 2 μg ml$^{-1}$ 5-FU (△–△)

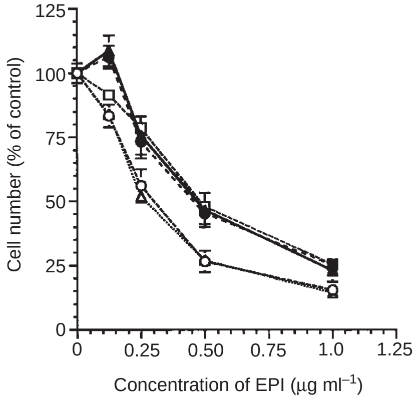

**Figure 7** The chemosensitivity of untreated (○–○) or treated CEM/A7R cells, 3 weeks after an overnight exposure to 0.5 μg ml$^{-1}$ of EPI (▲–▲), 0.1 μg ml$^{-1}$ of IDA (●–●) and MX2 (□–□), and 2 μg ml$^{-1}$ of 5-FU (△–△)

IDA and MX2 respectively (Figure 3). When the exposure time was prolonged up to 24 h, a fivefold increase of *MDR1* mRNA was observed at the $IC_{50}$ concentrations of IDA (0.02 μg ml$^{-1}$) or MX2 (0.05 μg ml$^{-1}$) (data not shown).

The potential induction of the *MDR1* gene by 5-FU was examined at 0.2, 0.5 and 2.0 μg ml$^{-1}$ ($IC_{10}$, $IC_{50}$ and $IC_{90}$) levels respectively. Although no obvious increase in *MDR1* expression was observed at any concentration of 5-FU after 4 h (data not shown), Northern analysis revealed induction had occurred in a dose-dependent manner after 24 h (Figure 4). A threefold induction in *MDR1* expression was seen at the $IC_{90}$ level (2 μg ml$^{-1}$) compared with the fivefold induction of *MDR1* at the $IC_{50}$ level (0.02 μg ml$^{-1}$) of IDA (Figure 4).

For each of the four drugs used in these experiments, treated cells were stained with trypan blue to assess cells for visible cell damage. Careful examination using phase-contrast microscopy for change in cell morphology and/or increased granularity revealed no visible evidence of cell damage within 4–24 h at any of the drug concentrations used.

### Pgp expression

To determine whether the increased levels of *MDR1* mRNA expression induced by IDA, MX2 and 5-FU were accompanied by corresponding changes in Pgp levels, antigen density (mean channel fluorescence or mcf) was measured by flow cytometry. The CEM/A7R cells were exposed for 24 h to approximately the $IC_{90}$ levels of IDA, MX2 (0.1 μg ml$^{-1}$), 5-FU (2.0 μg ml$^{-1}$) or EPI (0.5 μg ml$^{-1}$) before being subjected to Pgp analysis (Figure 5). In parallel to the increase in the *MDR1* mRNA levels (Figure 3), Pgp expression was increased five- to sevenfold with EPI, MX2 and IDA (mcf 36.65, 25.98 and 22.20 respectively) and threefold with 5-FU (mcf 18.74). In comparison, the mcf of untreated CEM/A7R cells was 4.2.

To examine whether the induction in *MDR1* gene expression was stable, CEM/A7R cells were treated overnight with 0.1 μg ml$^{-1}$ MX2 ($IC_{90}$) or 2.0 μg ml$^{-1}$ 5-FU ($IC_{90}$). The drug treated cells were then washed three times and allowed to grow in drug-free medium for either 72 h or 3 weeks before being subjected to a further analysis of *MDR1* mRNA levels (data not shown) or Pgp expression. In the MX2- and IDA-treated CEM/A7R cells, Pgp expression remained two- to threefold higher (mcf 13.50 and 11.09) than the untreated CEM/A7R cells (mcf 4.66) 3 weeks after drug exposure. In contrast, the increased Pgp levels in cells treated with 5-FU returned to base line (mcf 4.69) within 72 h of stopping drug treatment (data not shown).

To assess the functional activity of Pgp, [$^3$H]daunomycin accumulation was determined in the CEM/A7R cells. After an overnight exposure to EPI (0.5 μg ml$^{-1}$), IDA (0.1 μg ml$^{-1}$), MX2

© Cancer Research Campaign 1999

(0.1 μg ml–1) or 5-FU (2.0 μg ml–1), the cells were maintained in drug-free medium for 3 weeks before drug accumulation was quantified. Compared with the untreated CEM/A7R cells, the intracellular levels of [3H]daunomycin were significantly lower in the EPI-, IDA- and MX2-treated CEM/A7R cells, but were unchanged in the 5-FU-treated cells (Figure 6). Growth inhibition assays also showed a two- to threefold increase in resistance to EPI in MX2-, IDA- and EPI-treated cells after 3 weeks in drug-free medium. In contrast, the drug sensitivity of 5-FU-treated cells was the same as that of untreated CEM/A7R cells (Figure 7).

### DISCUSSION

In this report, we investigated whether the two lipid-soluble anthracycline analogues IDA and MX2 could up-regulate *MDR1* gene expression in a human MDR, T-cell leukaemia line, CEM/A7R, and its parental line, CCRF-CEM. Both drugs are thought to be poor substrates for Pgp-mediated transport (de Vries and Zijlstra, 1990; Ross et al, 1995). Initially, we compared the chemosensitivity (in the presence or absence of Vp or PSC 833) of each drug in the cell lines.

Verapamil (5 μM) and PSC 833 (1 μM) dramatically decreased the resistance of the CEM/A7R line to EPI, but had no effect on the sensitivity of this drug-resistant line to MX2 and only slightly increased the sensitivity to IDA (Figure 1). These findings are consistent with the observation that both anthracycline analogues are highly lipophilic and diffuse rapidly through the cell membrane to bind to DNA, thereby interfering with the capacity of cells expressing Pgp to pump these drugs (Horichi et al, 1990; Watanabe et al, 1991; Berman and McBride, 1992) and thus overcoming drug resistance. Although IDA and MX2 are less susceptible to this mechanism of resistance compared with the drug-sensitive line, detectable levels of resistance to IDA and MX2 were still observed in the resistant CEM/A7R cells (Table 1).

The effects of IDA, MX2 and 5-FU on the induction of *MDR1* gene expression were investigated at equieffective concentrations with respect to the inhibition of cell growth in a 72-h cell growth inhibition assay. When *MDR1* mRNA expression was analysed by Northern blotting, a two- to fivefold increase was observed in the CEM/A7R cells 4 h after treatment with IDA, MX2 or EPI at concentrations ranging from the $IC_{10}$ to $IC_{90}$ level. In contrast, 5-FU failed to up-regulate *MDR1* expression after a 4-h exposure, although induction of *MDR1* gene expression was seen after a 24-h exposure. The effects of these drugs on *MDR1* gene expression was confirmed by flow analysis, demonstrating a concomitant increase in Pgp expression. Up-regulation of Pgp expression by IDA and MX2 was stable, as demonstrated by the increase in Pgp levels, drug resistance and decrease in drug accumulation in the IDA- and MX2-treated CEM/A7R cells 3 weeks after the removal of these agents from the culture medium (Figures 5–7).

Other investigators have reported the up-regulation of *MDR1* promoter activity in reporter gene assays by cytotoxic drugs (Kohno et al, 1989) as well as many other factors (Rohlff and Glazer, 1995). These findings have been controversial with respect to their relevance in the clinical context, as the endogenous promoter does not always behave in an analogous manner to the transfected promoter. For example, Tanimura et al (1992) and Ferrandis and Benard (1993) have demonstrated that a transfected promoter is active in drug-sensitive cells in which the endogenous promoter is not active.

Increased *MDR1* mRNA and Pgp expression have also been reported in human cell lines after their exposure to cytotoxic drugs (Chaudhary and Roninson, 1993; Gekeler et al, 1994). However, the possibility that selection had occurred could not be excluded (reviewed in Manzano et al, 1996), as drug-induced *MDR1* expression usually occurred after a 72-h exposure to sublethal concentrations of cytotoxics and was associated with the appearance of visible morphological cell damage.

The present study was designed to examine the role of the two anthracycline analogues IDA and MX2 on the up-regulation of *MDR1* gene expression for a range of concentrations ($IC_{10}$, $IC_{50}$ and $IC_{90}$ levels) within 4 and 24 h. In our study, the increase in *MDR1* expression was not related to the appearance of cytological damage and was seen at concentrations as low as the $IC_{10}$ level for each drug.

Up-regulation of *MDR1* gene expression has mainly been observed for drugs which are substrates for Pgp, although one study demonstrated that a non-Pgp substrate, cytarabine, was able to induce *MDR1* expression (after a 72-h exposure) which was stable after withdrawal of the drug for more than 6 weeks (Chaudhary and Roninson, 1993). In this report, we observed that 5-FU, also not a substrate for Pgp, could up-regulate *MDR1* mRNA levels. However, this appeared to be a transient increase because the level of Pgp returned to baseline within 72 h of removal of the drug. These findings suggest that the mechanism by which 5-FU acts probably differs from that used by other anthracyclines which result in a sustained increase in Pgp expression.

It is likely that the cellular response to cytotoxic stress is to activate a number of defence mechanisms via stress response pathways (Osborn and Chambers, 1996). The up-regulation of Pgp expression may represent only one of the results of this response to these stress mechanisms. However, this finding would not be expected to confer resistance to IDA or other drugs that are not pumped by Pgp (Consul et al, 1996; Hargrave et al, 1995). Thus, the up-regulation of Pgp expression in response to such drugs does not necessarily confer a survival advantage. In fact, it is generally accepted that the treatment of relapsed refractory leukaemia with idarubicin/cytarabine is associated with higher rates of complete remission and longer survival compared with daunorubicin/cytarabine (Carella et al, 1993; Berman, 1993).

The mechanism by which these lipid-soluble anthracyclines, or indeed the classic Pgp substrate EPI, up-regulate Pgp expression is not known. The fact that induction can occur rapidly (in 4 h) strongly suggests that up-regulation involves transcription of the *MDR1* gene, perhaps involving stress response pathways. Osborn and Chambers (1996) demonstrated that a c-*jun* $NH_2$-terminal protein kinase (JNK), a member of the mitogen-activated protein kinase family, is activated by a variety of stressful stimuli, including exposure to cytotoxic agents. In their system, exposure to doxorubicin resulted in increased JNK activity and increased *MDR1* mRNA levels. Rohlff and Glazer (1995) suggested a number of signalling pathways may act on *MDR1* gene expression. Which, if any, of these pathways are involved in the induction response observed in our line is the focus of further work in our laboratory.

### ABBREVIATIONS

IDA, 4-demethoxydaunorubicin (Idarubicin); MX2, 3′-deamino-3′-morpholino-13-deoxo-10-hydroxycarminomycin; EPI, epirubicin; DOX, doxorubicin; 5-FU, 5-fluorouracil; CyA, cyclosporin A; Vp, verapamil; MDR, multidrug resistance; Pgp, P-glycoprotein; mcf, mean channel fluorescence.

© Cancer Research Campaign 1999

### ACKNOWLEDGEMENT

This study was supported in part by the Anti-Cancer Council of Victoria and Department of Veterans Affairs, Canberra, Australia.

### REFERENCES

Beketic-Oreskovic L, Duran GE, Chen G, Dumontet C and Sikic BI (1995) Decreased mutation rate for cellular resistance to doxorubicin and suppression of *mdr1* gene activation by the cyclosporin PSC 833. *J Natl Cancer Inst* **87**: 1593–1602

Berman E (1993) A review of idarubicin in acute leukemia. *Oncology* **7**: 91–104

Berman E and McBride M (1992) Comparative cellular pharmacology of daunorubicin and idarubicin in human multidrug-resistant leukemia cells. *Blood* **79** (1): 3267–3273

Bosch I and Croop J (1996) P-glycoprotein multidrug resistance and cancer. *Biochim Biophys Acta* **1288**: F37–F54

Carella AM, Carlier P, Pungolino E, Resegotti L, Liso V, Stasi R, Montillo M, Iacopino P, Mirto S, Pagano L, Leoni F, Martelli FM, Raimondo FD, Porcellini A, de Rui L, Nosari AM, Cimino R, Damasio E, Miraglia E, Fioritoni G, Ricciuti F, Carotenuto M, Longinotti M, Defazio D, Fazi P and Mandelli F (1993) Idarubicin in combination with intermediate-dose cytarabine and Vp-16 in the treatment of refractory or rapidly relapsed patients with acute myeloid leukemia. *Leukemia* **7**: 196–199

Chaudhary PM and Roninson IB (1993) Induction of multidrug resistance in human cells by transient exposure to different chemotherapeutic drugs. *J Natl Cancer Inst* **85**: 632–639

Chen G, Jaffrézou JP, Fleming WH, Durán GE and Sikie BI (1994) Prevalence of multidrug resistance related to activation of the *MDR1* gene in human sarcoma mutants derived by single-step doxorubicin selection. *Cancer Res* **54**: 4980–4987

Chin KV, Chauhan SS, Pastan I and Gottesman MM (1990) Regulation of mdr RNA levels in response to cytotoxic drugs in rodent cells. *Cell Growth Differ* **1**: 361–365

Chomczynski P and Sacchi N (1987) Single-step method of RNA isolation by acid guanidinium thiocyanate–phenol–chloroform extraction. *Anal Biochem* **162**: 156–159

Consoli U, Priebe W, Ling YH, Mahadevia R, Griffin M, Zhao S, Soler RP and Andreeff M (1996) The novel anthracycline annamycin is not affected by P-glycoprotein related multidrug resistance: comparison with idarubicin and doxorubicin in HL60 leukemia cell lines. *Blood* **88**: 633–644

de Vries EGE and Zijlstra JG (1990) Morpholinyl anthracyclines: option for reversal of anthracycline resistance. *Eur J Cancer* **26**: 659–660

Deuchars KL and Ling V (1989) P-glycoprotein and multidrug resistance in cancer chemotherapy. *Semin Oncol* **16**: 156–165

Dumontet C, Duran GE, Steger KA, Beketic-Oreskovic L and Sikic BI (1996) Resistance mechanisms in human sarcoma mutants derived by single-step exposure to paclitaxel (Taxol). *Cancer Res* **56**: 1091–1097

Fardel O, Lecureur V, Daval S, Corlu A and Guillouzo A (1997) Up-regulation of P-glycoprotein expression in rat liver cells by acute doxorubicin treatment. *Eur J Biochem* **246**: 186–192

Ferrandis E and Benard J (1993) Activation of the human *MDR1* gene promoter in differentiated neuroblasts. *Int J Cancer* **54**: 987–991

Gekeler V, Beck J, Noller A, Wilisch A, Fresc G, Neumann M, Handgretinger R, Ehninger G, Probst H, Neithammer D (1994) Drug-induced changes in the expression of *MDR*-associated gene: investigations on cultured cell lines and chemotherapeutically treated leukemias. *Ann Hematol* **69**: S19–S24

Grogan TM, Spier CM, Salmon SE, Matzner M, Rybski J, Weinstein RS, Scheper RJ and Dalton WS (1993) P-glycoprotein expression in human plasma cell myeloma: correlation with prior chemotherapy. *Blood* **81**: 490–495

Hargave RM, Davey MW, Davey RA, Kidman AD (1995) Development of drug resistance is reduced with idarubicin relative to other anthracyclines. *Anti-Cancer Drugs* **6**: 432–437

Horichi N, Tapiero H, Sugimoto Y, Bungo M, Nishiyama M, Fourcade A, Lampidis TJ, Kasahara K, Sasaki Y, Takahashi T and Saijo N (1990). 3′-Deamino-3′-morpholino-13-deoxo-10-hydroxycarminomycin conquers multidrug resistance by rapid influx following higher frequency of formation of DNA single- and double-strand breaks. *Cancer Res* **50**: 4698–4701

Hu XF, de Luise M, Martin TJ and Zalcberg JR (1990*b*) Effect of cyclosporin and verapamil on the cellular kinetics of daunorubicin. *Eur J Cancer* **26**: 814–817

Hu XF, Martin TJ, Bell DR, de Luise M and Zalcberg JR (1990*a*) Combined use of cyclosporin A and verapamil in modulating multidrug resistance in human leukemia cell lines. *Cancer Res* **50**: 2953–2957

Hu XF, Slater S, Wall DM, Kantharidis P, Parkin JD, Cowman A and Zalcberg JR (1995) Rapid upregulation of mdr1 expression by anthracyclines in a classical multidrug-resistant cell line. *Br J Cancer* **71**: 931–936

Hu XF, Slater A, Wall DM, Parkin JD, Kanthrarids P and Zalcberg JR (1996) Cyclosporin A and PSC 833 prevent up-regulation of *MDR1* expression by anthracyclines in a human multidrug-resistant cell line. *Clin Cancer Res* **2**: 713–720

Kohno K, Sato S, Takano H, Matsuo K-I and Kuwano M (1989) The direct activation of human multidrug resistance gene (*MDR1*) by anticancer agents. *Biochem Biophys Res Commun* **165**: 1415–1421

Manzano RG, Wright VK and Twentyman PR (1996) Rapid recovery of a functional MDR phenotype caused by MRP after a transient exposure to MDR drugs in a revertant human lung cancer cell line. *Eur J Cancer* **32A**: 2136–2141

Michieli M, Michelutti A, Damiani D, Pipan C, Paspadori D, Lauria F and Baccarani M (1993) A comparative analysis of the sensitivity of multidrug resistant (MDR) and non-MDR cells to different anthracycline derivatives. *Leukemia Lymphoma* **9**: 255–264

Mickenna SL and Padua RA (1997) Review: multidrug resistance in leukemia. *Br J Haematol* **96**: 659–674

Nielsen C, Maare C and Skovesgaard T (1996) Cellular resistance to anthracyclines. *Gen Pharmacol* **27**: 251–255

Nooter K and Sonneveld P (1994) Clinical relevance of P-glycoprotein expression in haematological malignancies. *Leukemia Res* **18**: 233–243

Osborn MT and Chambers TC (1996) Role of the stress-activated/c-*jun* $NH_2$-terminal protein kinase pathway in cellular response to adriamycin and other chemotherapeutic drugs. *J Biol Chem* **271**: 30950–30955

Poeta GD, Stasi R, Aronica G, Venditti A, Cox MC, Bruno A, Buccisano F, Masi M, Tribalto M, Amadori S and Papat G (1996) Clinical relevance of P-glycoprotein expression in de novo acute myeloid leukemia. *Blood* **87**: 1997–2004

Rohlff C and Glazer RI (1995) Regulation of multidrug resistance through the c-AMP and EGF signalling pathways. *Cell Signalling* **7**: 431–443

Ross DD, Doyle LA, Yang W, Tong Y and Cornblatt B (1995) Susceptibility of idarubicin, daunorubicin and their C-13 alcohol metabolites to transport-mediated multidrug resistance. *Biochem Pharmacol* **50**: 1673–1683

Rothenberg M, Mickley LA, Cole DE, Balis FM, Tsuruo T, Poplack D and Fojo AT (1989) Expression of the *mdr1*/*P-170* gene in patients with acute lymphoblastic leukemia. *Blood* **74**: 1388–1395

Shustik C, Dalton W and Gros P (1995) P-glycoprotein-mediated multidrug resistance in tumor cells; biochemistry, clinical relevance and modulations. In *Molecular Aspects of Medicine*, pp. 1–78. Elsvier Science: London

Tanimura H, Kohno K, Sato S-i, Uchiumi T, Miyazaki M, Kobayashi M and Kuwano M (1992) The human multidrug resistance 1 promoter has an element that responds to serum starvation. *Biochem Biophys Comm* **183**: 917–924

Tsuruo T, Iida H, Tsukagoshi S and Sakurai Y (1981) Overcoming of vincristine resistance in P388 leukemia in vivo and in vitro through enhanced cytotoxicity of vincristine and vinblastine by verapamil. *Cancer Res* **41**: 1967–1972

Ueda K, Clark DP, Chen C-J, Roninson IB, Gottesman MM and Pastan I (1987) The human multidrug resistance (*mdr1*) gene: cDNA cloning and transcription initiation. *J Biol Chem* **262**: 505–508

Watanabe M, Komeshima N, Naito M, Isoe T, Otake N and Tsuruo T (1991) Cellular pharmacology of MX2, a new morpholino anthracycline, in human pleiotropic drug-resistant cells. *Cancer Res* **51**: 157–161

Zalcberg JR, Hu XF, Wall DM, Mirski S, Cole S, Nadalin G, De Luise M, Parkin JD, Vrazas V, Campbell L and Kantharidis P (1994) Cellular and karyotypic characterization of two doxorubicin resistant cell lines isolated from the same parental human leukemia cell line. *Int J Cancer* **57**: 522–528

© Cancer Research Campaign 1999
